# Correlated bursts in temporal networks slow down spreading

**DOI:** 10.1038/s41598-018-33700-8

**Published:** 2018-10-17

**Authors:** Takayuki Hiraoka, Hang-Hyun Jo

**Affiliations:** 1 Asia Pacific Center for Theoretical Physics, Pohang, 37673 Republic of Korea; 2 Department of Physics, Pohang University of Science and Technology, Pohang, 37673 Republic of Korea; 3 Department of Computer Science, Aalto University, Espoo, FI-00076 Finland

## Abstract

Spreading dynamics has been considered to take place in temporal networks, where temporal interaction patterns between nodes show non-Poissonian bursty nature. The effects of inhomogeneous interevent times (IETs) on the spreading have been extensively studied in recent years, yet little is known about the effects of correlations between IETs on the spreading. In order to investigate those effects, we study two-step deterministic susceptible-infected (SI) and probabilistic SI dynamics when the interaction patterns are modeled by inhomogeneous and correlated IETs, i.e., correlated bursts. By analyzing the transmission time statistics in a single-link setup and by simulating the spreading in Bethe lattices and random graphs, we conclude that the positive correlation between IETs slows down the spreading. We also argue that the shortest transmission time from one infected node to its susceptible neighbors can successfully explain our numerical results.

## Introduction

Characterizing the interaction structure of constituents of complex systems is of utmost importance to understand the dynamical processes in those systems. The interaction structure has been modeled by a network, where nodes and links denote the constituents and their pairwise interactions, respectively^1,2^. When the interactions are temporal, one can adopt a framework of temporal networks^3^, where links are considered to exist only at the moment of interaction. Events in the temporal interaction patterns are often known to be non-Poissonian or bursty^4–6^, e.g., as shown in human communication patterns^7–14^. Here, bursts denote a number of events occurring in short active periods separated by long inactive periods, which can be related to 1/*f* noise^15–17^. In general, non-Poissonian temporal patterns can be fully understood both by inhomogeneous interevent times (IETs) and by correlations between IETs^18^. Inhomogeneous and correlated IETs have been called *correlated bursts*^5,19^. Then, along with the information on who interacts with whom, one can comprehensively characterize the topological and temporal interaction structure of complex systems^20^.

Non-Poissonian bursty interactions between constituents of a system have been known to strongly affect the dynamical processes taking place in the system^21–32^; in particular, spreading dynamics in temporal networks has been extensively studied. An important question is what features of temporal networks are most relevant to predict the speed of propagation, e.g., of disease or information. One of the crucial and widely studied features is the inhomogeneity of IETs in the temporal interaction patterns. It was shown that the bursty interaction patterns can slow down the early-stage spreading by comparing the simulated spreading behaviors in some empirical networks and in their randomized versions^21,23,29^. The opposite tendency was also reported using another empirical network or model networks^25,26,30^.

In contrast to the IET distributions, yet little is known about the effects of correlations between IETs on the spreading, except for few recent works^33,34^. This could be partly because many previous works have considered the contagion dynamics, e.g., susceptible-infected (SI) dynamics^31^, with the assumption of an immediate infection upon the first contact between susceptible and infected nodes, hence without the need to consider correlated IETs. In another work^35^, probabilistic contagion dynamics, i.e., naturally involving multiple consecutive IETs, was studied by assuming inhomogeneous but uncorrelated IETs. Therefore, the effects of inhomogeneous and correlated IETs on the spreading need to be systematically studied for better understanding the dynamical processes in complex systems.

In our paper, we study the effects of inhomogeneous and correlated IETs on the spreading taking place in temporal networks, by incorporating two contagion models, i.e., two-step deterministic SI and probabilistic SI dynamics. For modeling the inhomogeneous IETs, we consider power-law distributions of IETs, denoted by *τ*, as

1 $$P(\tau )\sim {\tau }^{-\alpha },$$

with power-law exponent *α*^6^. For characterizing the correlations between IETs, we adopt a memory coefficient *M*^36^ among others, e.g., bursty train sizes^5^. The memory coefficient *M* for a sequence of *n* IETs has been estimated by

2 $$M\equiv \frac{1}{n-1}\sum _{i=1}^{n-1}\,\frac{({\tau }_{i}-{\mu }_{1})({\tau }_{i+1}-{\mu }_{2})}{{\sigma }_{1}{\sigma }_{2}},$$

where *μ*_1_ (*μ*_2_) and *σ*_1_ (*σ*_2_) are the average and the standard deviation of the first (last) *n*−1 IETs, respectively. Positive *M* implies the tendency of large (small) IETs being followed by large (small) IETs. Negative *M* points towards the opposite, while *M* = 0 for uncorrelated IETs. In our setup, both *P*(*τ*) and *M* are inputs of the model, requiring us to consider *M* as a parameter rather than an estimator. Then by controlling the shape of *P*(*τ*) and the value of *M* for interaction patterns between nodes, one can study the effects of correlated bursts on the spreading in temporal networks. By analyzing the contagion dynamics on a single link, and then by simulating the spreading in regular and random temporal networks, we conclude that the positively-correlated inhomogeneous IETs slow down the spreading.

## Models

**Figure 1:**
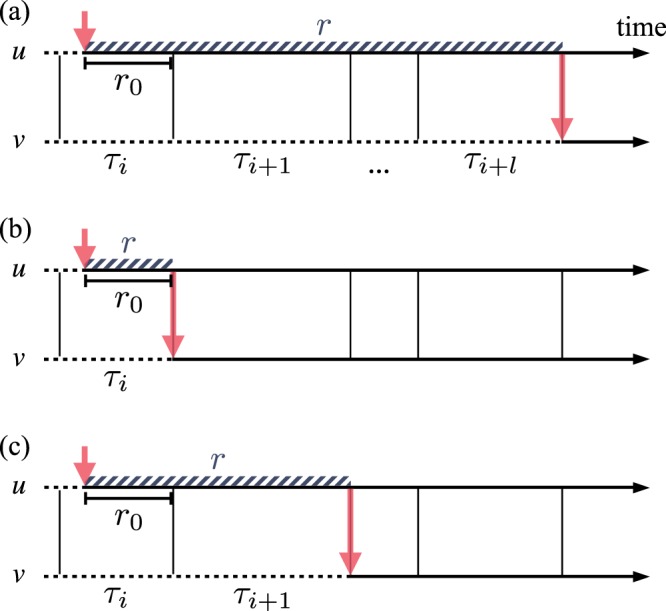
Schematic diagrams for (**a**) the probabilistic susceptible-infected (SI) dynamics, (**b**) the one-step deterministic SI dynamics, and (**c**) the two-step deterministic SI dynamics. For each node, the susceptible or intermediate state is represented by a dashed horizontal line, while the infected state is by a solid horizontal line. In each panel, a node *u* gets infected in the time denoted by an upper vertical arrow, then it tries to infect its susceptible neighbor *v* whenever they make contact (vertical lines). The successful infection of *v* by *u* is marked by a lower vertical arrow. The time interval between the infection of *u* and that of *v* (striped band) defines the transmission time *r*. For the definitions of *r*_0_ and τs, see the text.

In order to study the spreading dynamics, we consider one of the extensively studied epidemic processes, i.e., susceptible-infected (SI) dynamics^31^: A state of each node in a network is either susceptible (*S*) or infected (*I*), and an infected node can infect a susceptible node by the contact with it. Here we assume that the contact is instantaneous. One can study a probabilistic SI (PSI) dynamics, in which an infected node can infect a susceptible node with probability *η* (0 < *η* < 1) per contact, as depicted in Fig. 1(a), i.e.

$$S+I\,\mathop{\longrightarrow }\limits^{\eta }\,2I\mathrm{.}$$

Due to the stochastic nature of infection, multiple IETs can be involved in the contagion, hence the correlations between IETs in the contact patterns can influence the spreading behavior. The case with *η* = 1 corresponds to the deterministic version of SI dynamics, which we call one-step deterministic SI (1DSI) dynamics: A susceptible node is immediately infected after its first contact with an infected node, see Fig. 1(b). This dynamics can be described by

$$S+I\to 2I.$$

Finally, for studying the effect of correlations between IETs on the spreading in a simpler setup, we introduce two-step deterministic SI (2DSI) dynamics as a variation of generalized epidemic processes^37–40^, see Fig. 1(c). Here a susceptible node first changes its state to an intermediate state (*S*′) upon its first contact with an infected node; it then becomes infected after the second contact with the same or another infected node. This can be written as

$$\begin{array}{rcl}S+I & \to  & S^{\prime} +I,\\ S^{\prime} +I & \to  & 2I\mathrm{.}\end{array}$$

For modeling the interaction structure in a population, we focus on Bethe lattices as networks of infinite size, while regular random graphs and Erdös-Rényi random graphs will be later considered for finite network models. As for the temporal contact patterns, we assume that the contacts between a pair of nodes or on a link connecting these nodes are instantaneous and undirected. Moreover, the contact pattern on each link is assumed to be independent of the states of two end nodes as well as of contact patterns on other links. The contact pattern on each link is modeled by a statistically identical event sequence with inhomogeneous and correlated IETs. For this, the shape of IET distribution *P*(*τ*) and the value of memory coefficient *M* are inputs of our model. Firstly, we consider a power-law IET distribution with a lower bound *τ*_min_ and an exponential cutoff *τ*_c_ as follows:

3 $$P(\tau )=(\begin{array}{ll}\frac{{\tau }_{{\rm{c}}}^{\alpha -1}}{{\rm{\Gamma }}\mathrm{(1}-\alpha ,{\tau }_{{\rm{\min }}}/{\tau }_{{\rm{c}}})}{\tau }^{-\alpha }{e}^{-\tau /{\tau }_{{\rm{c}}}} & {\rm{for}}\,\tau \ge {\tau }_{{\rm{\min }}},\\ 0 & {\rm{otherwise}},\end{array}$$

where Γ is the upper incomplete Gamma function. We fix *τ*_min_ = 1 without loss of generality and set *τ*_c_ = 10^3^ in our work. We note that our conclusions are robust with respect to the choice of *τ*_c_. Thus, we are left with one relevant parameter, i.e., the power-law exponent *α*. Based on the empirical findings for *α*^6^, we consider the case with 1.5 ≤ *α* ≤ 3. Secondly, only the positive memory coefficient *M* is considered, precisely, 0 ≤ *M* < 0.4, based on the empirical observations^36,41–43^.

## Results

### Two-step deterministic contagion

#### Single-link transmission

For understanding the spreading behavior on temporal networks, we first focus on how long it takes for the infection to transmit across a single link, say, from a node *u* to its neighbor *v*. If *u* gets infected from its neighbor other than *v* in time *t*_*u*_, and later it infects *v* in time *t*_*v*_, the time interval between *t*_*u*_ and *t*_*v*_ defines the transmission time *r* ≡ *t*_*v*_ − *t*_*u*_. Here we assume that *v* is not affected by any other neighbors than *u*, for the sake of simplicity. In order for the infected *u* to infect the susceptible *v*, *u* must wait at least for the next contact with *v*. This waiting or residual time is denoted by *r*_0_, see Fig. 1. For the one-step deterministic SI dynamics, *r* = *r*_0_. Due to the independence of contact patterns of neighboring links, we can consider the infection of *u* to occur at random in time. In addition, since only the IETs larger than *r* contribute to the probability of having the transmission time *r*, we have the transmission time distribution as

4 $${Q}_{1{\rm{D}}}(r)=\frac{1}{\mu }{\int }_{r}^{\infty }\,d\tau P(\tau ),$$

with *μ* denoting the mean IET, see Methods for the detailed derivation. The average transmission time is directly obtained as

5 $${\langle r\rangle }_{{\rm{1}}D}\equiv {\int }_{0}^{\infty }\,drr{Q}_{1{\rm{D}}}(r)=\frac{1}{2}(\mu +\frac{{\sigma }^{2}}{\mu }),$$

where *σ*^2^ denotes the variance of IETs^6^. Note that a larger variance of IETs results in a larger average transmission time, expected to slow down the spreading.

In general, *r* can be given as the sum of *r*_0_ and subsequent IETs, as depicted in Fig. 1(a,c), for the generalized epidemic processes, including our two-step deterministic SI (2DSI) dynamics. In the case with 2DSI dynamics, the transmission process involves two consecutive IETs. If the infection of *u* occurs during the IET of *τ*_*i*_, then the transmission time is written as

6 $$r={r}_{0}+{\tau }_{i+1},$$

with *τ*_*i* +1_ denoting the IET following *τ*_*i*_. Information on the correlations between *τ*_*i*_ and *τ*_*i*+1_ is carried by the joint distribution *P*(*τ*_*i*_, *τ*_*i*+1_) or the conditional distribution *P*(*τ*_*i* +1_|*τ*_*i*_). Using *P*(*τ*_*i* +1_|*τ*_*i*_) with *τ*_*i* +1_ = *r* − *r*_0_, *Q*_1D_(*r*) in equation (4) can be extended to obtain the transmission time distribution for the 2DSI case as

7 $${Q}_{{\rm{2}}D}(r)=\frac{1}{\mu }{\int }_{0}^{r}\,d{r}_{0}{\int }_{{r}_{0}}^{\infty }\,d{\tau }_{i}P({\tau }_{i})P(r-{r}_{0}|{\tau }_{i}),$$

where it is obvious from equation (6) that *τ*_*i*_ ≥ *r*_0_ and 0 ≤ *r*_0_ ≤ *r*, see Methods for the detailed derivation. The average transmission time is calculated as

8 $${\langle r\rangle }_{2{\rm{D}}}\equiv {\int }_{0}^{\infty }\,drr{Q}_{2{\rm{D}}}(r)={\langle r\rangle }_{1{\rm{D}}}+\frac{1}{\mu }\langle {\tau }_{i}{\tau }_{i+1}\rangle ,$$

where

9 $$\langle {\tau }_{i}{\tau }_{i+1}\rangle \equiv {\int }_{0}^{\infty }\,d{\tau }_{i}{\int }_{0}^{\infty }\,d{\tau }_{i+1}{\tau }_{i}{\tau }_{i+1}P({\tau }_{i},{\tau }_{i+1}\mathrm{).}$$

In order to relate this result to the memory coefficient in equation (2), we define a parameter as

10 $$M\equiv \frac{\langle {\tau }_{i}{\tau }_{i+1}\rangle -{\mu }^{2}}{{\sigma }^{2}}$$

to finally obtain the analytical result of the average transmission time:

11 $${\langle r\rangle }_{{\rm{2}}D}=\frac{3}{2}\mu +(\frac{1}{2}+M)\frac{{\sigma }^{2}}{\mu }\mathrm{.}$$

In the case with *M* = 0 for uncorrelated IETs, one gets 〈*r*〉_2D_ = 〈*r*〉_1D_ + *μ*.

We remark that our result in equation (11) is valid for arbitrary functional forms of IET distributions as long as their mean and variance are finite. *M* is coupled with *σ*^2^/*μ*, implying that the impact of correlations between IETs becomes larger with broader IET distributions. More importantly, we find that a stronger positive correlation between consecutive IETs leads to a larger average transmission time. This can be understood in terms of the role of the variance of IETs in the average transmission time, as shown in the 1DSI case. That is, the variance of the sum of two consecutive IETs is amplified by the positive correlation between those IETs. Based on the result of the single-link analysis, the positive correlation between IETs is expected to slow down the spreading in a population.

#### Spreading in Bethe lattices

**Figure 2:**
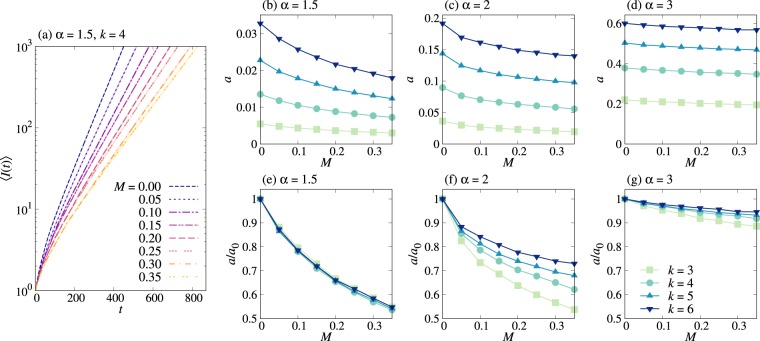
Two-step deterministic SI dynamics in Bethe lattices. (**a**) Average numbers of infected nodes as a function of time, 〈*I*(*t*)〉, in Bethe lattices with *k* = 4 for the same IET distribution with power-law exponent *α* = 1.5 in equation (3), but with various values of memory coefficient *M*. For each value of *M*, the average (dashed curve) and its standard error (shaded area) were obtained from 10^3^ runs with different initial conditions. (**b**–**g**) Estimated exponential growth rates *a*, defined in equation (12) (top panels) and their relative growth rates *a*/*a*_0_ with *a*_0_ ≡ *a*(*M* = 0) (bottom panels) are plotted for various values of *k*, *α*, and *M*. The lines are guides to the eye.

In order to investigate the effects of correlations between IETs on the spreading in a population, we numerically study spreading dynamics in a Bethe lattice, i.e., a regular tree of infinite size in which every node has exactly *k* neighbors. One can relate this dynamics to the early-stage dynamics in regular random graphs, in which cycles are rare as long as the network size is sufficiently large. As mentioned, the contact pattern on each link is modeled by an independent and identical point process with the same *P*(*τ*) and *M*. Precisely, for each link, we draw *n* random values from *P*(*τ*) to make an IET sequence *T* ≡ {*τ*_*i*_}_*i* = 1, …,*n*_, for sufficiently large *n*. Using equation (2), we measure the memory coefficient from *T*, denoted by $\tilde{M}$. Two IETs are randomly chosen in *T* and swapped only when this swapping makes $\tilde{M}$ closer to *M*, i.e., the target value. By repeating the swapping, we obtain the IET sequence whose $\tilde{M}$ is close enough to *M*, and from this IET sequence we get the sequence of contact timings for each link. Then the temporal network can be fully described by a set of contact timings for all links. Each simulation begins with one node infected at random in time, which we set as *t* = 0, while all other nodes are susceptible at this moment. For each simulation, we measure the number of infected nodes as a function of time, *I*(*t*). The average number of infected nodes 〈*I*(*t*)〉 is found to exponentially increase with time, e.g., as shown in Fig. 2(a):

12 $$\langle I(t)\rangle \sim {e}^{at},$$

where *a* = *a*(*k*, *α*, *M*) denotes the exponential growth rate, known as the Malthusian parameter^44^. *a* turns out to be a decreasing function of *M*, indicating the slowdown of spreading due to the positive correlation between IETs, see Fig. 2(b–d). The slowdown can be more clearly presented in terms of the relative growth rate *a*/*a*_0_ with *a*_0_ ≡ *a*(*M* = 0) for all cases of *k* and *α*, as shown in Fig. 2(e–g). We summarize the main observations from the numerical simulations as follows:

(i) *a* decreases with *M*.
 (ii) *a* increases with *α*.
(iii) *a* increases with *k*.
(iv) The deviation of *a*/*a*_0_ from 1 tends to be larger for smaller *α*.

The observation (i) is expected from equation (11), so is (ii) as both *μ* and *σ*^2^/*μ* decrease with *α*. (iii) is trivial. (iv) implies that the effect of *M* becomes larger for smaller *α*, which can be roughly understood by a larger value of *σ*^2^/*μ* coupled to *M* in equation (11). We remark that equation (11) is the result for a single-link transmission, requiring us to study the transmission time in networks.

**Figure 3:**
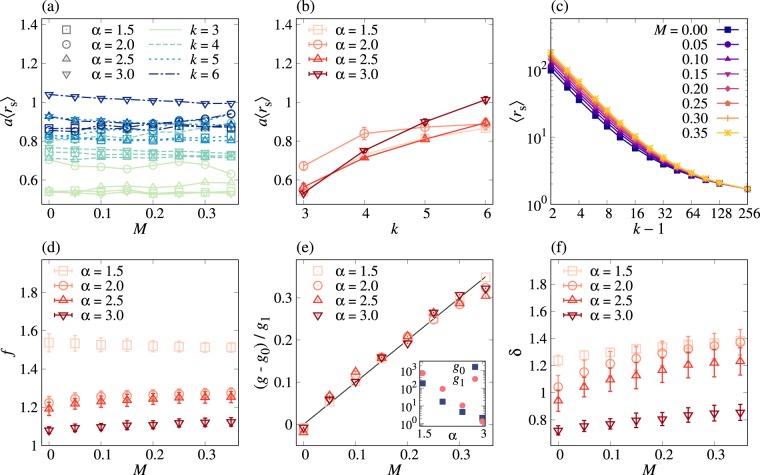
Two-step deterministic SI dynamics in Bethe lattices: The exponential growth rate *a* can be explained in terms of the average shortest transmission time 〈*r*_s_〉, where *r*_s_ is defined in equation (13). (**a**) *a*〈*r*_s_〉 is overall independent of *M* for all values of *k* and *α*. (**b**) *a*〈*r*_s_〉, averaged over *M*, is an increasing function of *k*. The error bars represent standard deviation. (**c**) For the case of *α* = 1.5, 〈*r*_s_〉 is plotted as a function of *k* − 1 for various values of *M*. Each point was averaged over 5 × 10^5^ realizations. In (**a**–**c**), the lines are guides to the eye. (**d**–**f**) Using a functional form of 〈*r*_s_〉 = *f* + *g*(*k* − 1)^−*δ*^ in equation (15), *f*, *g*, and *δ* are estimated for all values of *α* and *M*. In (**e**), using *g* = *g*_0_ + *g*_1_*M*, we estimate *g*_0_ and *g*_1_ and plot (*g* − *g*_0_)/*g*_1_ against *M*, compared to the black line of *y* = *x*. The estimated *g*_0_ and *g*_1_ are shown in the inset of (**e**).

In order to comprehensively understand the above observations, in particular, the *k*-dependence of *a*, we need to study the effect of time-ordering between infections to different neighbors^45^. For this, we introduce the shortest transmission time as

13 $${r}_{{\rm{s}}}\equiv \,{\rm{\min }}\,\{{r}^{\mathrm{(1)}},\,\cdots ,\,{r}^{(k-\mathrm{1)}}\},$$

where *r*^(*j*)^ for *j* = 1, …, *k* − 1 denotes the transmission time from an infected node to its *j*th neighbor. Here we focus on the average of *r*_s_, denoted by 〈*r*_s_〉, which is a function of *k*, *α*, and *M*. In Fig. 3(a), we numerically find that *a*〈*r*_s_〉 is independent of *M*, implying that the effect of the correlations between IETs on spreading can be fully understood by 〈*r*_s_〉. Then we write *a* as follows:

14 $$a=\frac{h(k,\alpha )}{\langle {r}_{{\rm{s}}}\rangle }\mathrm{.}$$

Here *h*(*k*, *α*) is generally expected to be a function of *k* and *α*, while only its *k*-dependence is clearly shown in Fig. 3(b), where *h*(*k*, *α*) increases with *k*. In Fig. 3(c) we observe that as *k* increases, 〈*r*_s_〉 algebraically decays before converging to a constant, enabling us to assume that

15 $$\langle {r}_{{\rm{s}}}\rangle =f+g{(k-\mathrm{1)}}^{-\delta },$$

where *f*, *g*, and *δ* are non-negative constants independent of *k*. By fitting the numerical results of 〈*r*_s_〉 using equation (15), we find how these constants depend on *α* and *M*, as summarized in Fig. 3(d–f).

Firstly, we find that *f* is overall independent of *M*. In the limit of *k* → ∞, 〈*r*_s_〉 should asymptotically approach the smallest possible transmission time, denoted by *r*_min_, leading to *f* = *r*_min_. For the 2DSI dynamics and by our setup, *f* = *τ*_min_ = 1 is expected, while the estimated values of *f* show systematic deviations from 1, possibly due to finite-size effects of *k*. Secondly and most importantly, *g* turns out to linearly increase with *M* such that

16 $$g={g}_{0}+{g}_{1}M,$$

with positive coefficients *g*_0_ and *g*_1_, eventually leading to the linear dependence of 〈*r*_s_〉 in equation (15) on *M*. Moreover, both *g*_0_ and *g*_1_ are found to decrease with *α*, shown in the inset of Fig. 3(e). These findings are comparable to the analytical result of average transmission time in equation (11). Finally, the estimated values of *δ* appear to slightly increase with *M*, while we consider *δ* to be constant of *M* in our argument. In sum, we rewrite 〈*r*_s_〉 in equation (15) as

17 $$\langle {r}_{{\rm{s}}}\rangle ={r}_{{\rm{\min }}}+({g}_{0}+{g}_{1}M)(k-{\mathrm{1)}}^{-\delta }\mathrm{.}$$

Combining *a* in equation (14) and 〈*r*_s_〉 in equation (17), we obtain the relative growth rate *a*/*a*_0_ as

18 $$\frac{a}{{a}_{0}}=\frac{{r}_{{\rm{\min }}}+{g}_{0}{(k-\mathrm{1)}}^{-\delta }}{{r}_{{\rm{\min }}}+({g}_{0}+{g}_{1}M)(k-{\mathrm{1)}}^{-\delta }},$$

by which the observation (iv) can be understood: In one limiting case when ${r}_{{\rm{\min }}}\ll {g}_{0}{(k-\mathrm{1)}}^{-\delta }$, the relative growth rate is approximated as

19 $$\frac{a}{{a}_{0}}\approx \frac{{g}_{0}}{{g}_{0}+{g}_{1}M},$$

which is independent of *k* but clearly showing the *M*-dependence. This can explain the numerical findings in the case with small *α* in Fig. 2(e). In the other limiting case when ${r}_{{\rm{\min }}}\gg ({g}_{0}+{g}_{1}M)(k-{\mathrm{1)}}^{-\delta }$, one gets

20 $$\frac{a}{{a}_{0}}\approx 1-\frac{{g}_{1}M}{{r}_{{\rm{\min }}}}{(k-\mathrm{1)}}^{-\delta },$$

i.e., *a*/*a*_0_ linearly but slightly decreases with *M*, showing a good agreement with the numerical results for large *α* in Fig. 2(g).

Conclusively, it turns out that the statistical properties of the shortest transmission time can account for the numerical results, while more refined approach needs to be taken for better understanding the effect of network structure on spreading, e.g., *k*-dependence of *a* in the case of Bethe lattices.

### Probabilistic contagion

#### Single-link transmission

In a more realistic scenario than the two-step deterministic contagion dynamics, the infection can be described by a stochastic process, i.e., probabilistic SI (PSI) dynamics: An infected node infects a susceptible node with probability *η* upon contact. Similarly to the deterministic cases, we begin with the analysis for a single-link transmission. If the infection of *u* occurs during the IET of *τ*_*i*_ as depicted in Fig. 1(a), the transmission time for a successful infection of *v* after *l* failed attempts for *l* ≥ 0 is

21 $$r=(\begin{array}{ll}{r}_{0} & {\rm{for}}\,l=\mathrm{0,}\\ {r}_{0}+\sum _{j=1}^{l}\,{\tau }_{i+j} & {\rm{for}}\,l > 0.\end{array}$$

Information on the correlations between *τ*_*i*_, …, *τ*_*i*+*l*_ is carried by the joint distribution *P*(*τ*_*i*_, …, *τ*_*i*+*l*_) for *l* + 1 consecutive IETs. The distribution of *r*, denoted by *Q*_P_(*r*), can be written as the weighted sum of transmission time distributions for multi-step deterministic dynamics, similarly done in the previous work^35^:

22 $${Q}_{{\rm{P}}}(r)=\sum _{l\mathrm{=0}}^{\infty }\,\eta {\mathrm{(1}-\eta )}^{l}{Q}_{l}(r),$$

where *Q*_*l*_(*r*) denotes the distribution of transmission time after *l* failed attempts. Note that *Q*_0_(*r*) = *Q*_1D_(*r*) in equation (4) and *Q*_1_(*r*) = *Q*_2D_(*r*) in equation (7). *Q*_*l*_(*r*) for general *l* ≥ 1 is obtained using the joint distribution *P*(*τ*_*i*_, …, *τ*_*i* +*l*_) as

23 $${Q}_{l}(r)=\frac{1}{\mu }{\int }_{0}^{r}\,d{r}_{0}\prod _{j\mathrm{=1}}^{l}{\int }_{0}^{\infty }\,d{\tau }_{i+j}{\int }_{{r}_{0}}^{\infty }\,d{\tau }_{i}P({\tau }_{i},\,\cdots ,\,{\tau }_{i+l})\delta (r-{r}_{0}-\sum _{j\mathrm{=1}}^{l}\,{\tau }_{i+j}),$$

where it is obvious from equation (21) that *τ*_*i*_ ≥ *r*_0_ and 0 ≤ *r*_0_ ≤ *r*, and *δ* denotes a Dirac delta function. Then one gets the average transmission time as follows:

24 $$\langle {r}_{l}\rangle \equiv {\int }_{0}^{\infty }\,drr{Q}_{l}(r)={\langle r\rangle }_{1{\rm{D}}}+\frac{1}{\mu }\sum _{j\mathrm{=1}}^{l}\,\langle {\tau }_{i}{\tau }_{i+j}\rangle ,$$

where

25 $$\langle {\tau }_{i}{\tau }_{i+j}\rangle \equiv \prod _{j^{\prime} =i}^{i+j}\,{\int }_{0}^{\infty }\,d{\tau }_{j^{\prime} }{\tau }_{i}{\tau }_{i+j}P({\tau }_{i},\,\cdots ,\,{\tau }_{i+j}\mathrm{).}$$

For the details of the derivation, see Methods. We define the generalized memory coefficient between two IETs separated by *j* − 1 IETs^36^ as

26 $${M}_{j}\equiv \frac{\langle {\tau }_{i}{\tau }_{i+j}\rangle -{\mu }^{2}}{{\sigma }^{2}},$$

leading to

27 $$\langle {r}_{l}\rangle ={\langle r\rangle }_{1{\rm{D}}}+l\mu +\frac{{\sigma }^{2}}{\mu }\sum _{j\mathrm{=1}}^{l}\,{M}_{j}\mathrm{.}$$

We then obtain the analytical result of the average transmission time for the PSI dynamics as

28 $$\begin{array}{rcl}{\langle r\rangle }_{{\rm{P}}} & \equiv  & {\int }_{0}^{\infty }\,drr{Q}_{{\rm{P}}}(r)=\sum _{l\mathrm{=0}}^{\infty }\,\eta {\mathrm{(1}-\eta )}^{l}\langle {r}_{l}\rangle \\  & = & {\langle r\rangle }_{1{\rm{D}}}+\frac{1-\eta }{\eta }\mu +\frac{{\sigma }^{2}}{\mu }\sum _{l=0}^{\infty }\,\eta {\mathrm{(1}-\eta )}^{l}\sum _{j=1}^{l}\,{M}_{j}\mathrm{.}\end{array}$$

As we introduce only the correlations between two consecutive IETs in our model, we expect *M*_*j*_ to exponentially decay according to *j*, where the decaying coefficient is denoted by *γ* with |*γ*| < 1: *M*_*j*_ = *γM*_*j*−1_ = … = *γ*^*j*−1^*M*_1_, where *M*_1_ = *M* in equation (10). Finally, we have

29 $${\langle r\rangle }_{{\rm{P}}}=(\frac{1}{2}+\frac{1-\eta }{\eta })\mu +[\frac{1}{2}+\frac{M\mathrm{(1}-\eta )}{1-\gamma \mathrm{(1}-\eta )}]\frac{{\sigma }^{2}}{\mu }\mathrm{.}$$

We note that this result is valid for arbitrary functional forms of IET distributions as long as their mean and variance are finite. Similarly to the deterministic case in equation (11), the average transmission time for the PSI case turns out to be a linearly increasing function of the memory coefficient *M*.

#### Spreading in Bethe lattices

**Figure 4:**
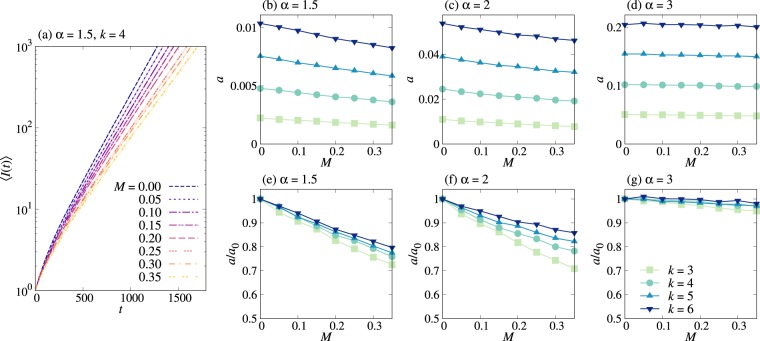
Probabilistic SI dynamics with *η* = 0.1 in Bethe lattices. All notations and simulation details are the same as in Fig. 2.

**Figure 5:**
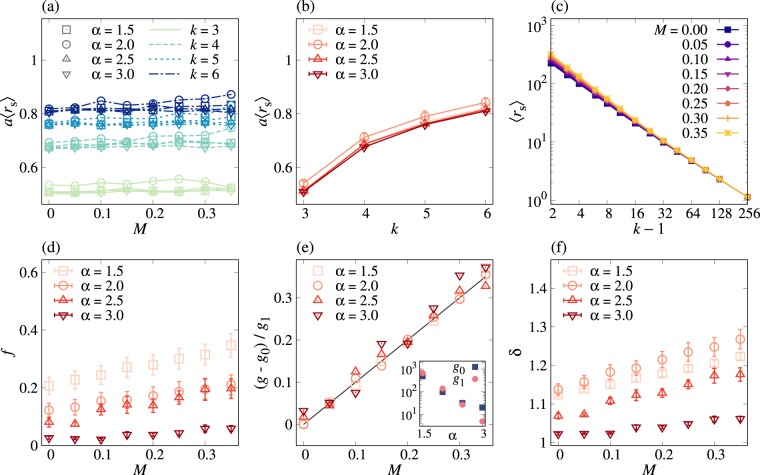
Probabilistic SI dynamics with *η* = 0.1 in Bethe lattices. All notations and simulation details are the same as in Fig. 3.

Next, we numerically examine the spreading behavior for the PSI dynamics with *η* < 1 in Bethe lattices. Similarly to the two-step deterministic case, we observe an exponential growth in the average number of infected nodes as well as the slowdown of spreading when the memory coefficient is positive. For example, the case with *η* = 0.1 is depicted in Fig. 4. As *η* approaches 1, the slowdown effect due to the correlated IETs becomes weak, as expected (not shown). Based on the results in Fig. 5, we make overall the same conclusions as in the 2DSI case: *a* is a decreasing (increasing) function of *M* (both *α* and *k*), and the deviation of *a*/*a*_0_ from 1 tends to be larger for smaller *α*.

The above observations in the PSI case can be understood by the same argument made for the 2DSI case, namely, the functional form of *a* in equation (14) with 〈*r*_s_〉 in equation (17), but with some important differences: Firstly, the shortest possible transmission time is *r*_min_ = 0, although the estimated values of *f* show systematic deviations from 0 in Fig. 5(d). This deviation is denoted by a small positive value *ε*, leading to *f* = *ε*. Secondly, *δ* appears to be an increasing function of *M* rather than a constant in Fig. 5(f), which we assume to be *δ* = *δ*_0_ + *δ*_1_*M* with positive coefficients *δ*_0_ and *δ*_1_. We therefore modify 〈*r*_s_〉 in equation (17) as follows:

30 $$\langle {r}_{{\rm{s}}}\rangle =\varepsilon +({g}_{0}+{g}_{1}M)(k-{\mathrm{1)}}^{-({\delta }_{0}+{\delta }_{1}M)}\mathrm{.}$$

We note that due to the positive *δ*_1_, the above 〈*r*_s_〉 may decrease with *M* but only for sufficiently large *k* and *M*. However, we find no evidence for the decreasing behavior in the ranges of *k* and *M* studied in our paper. Using equation (30), the relative growth rate is obtained as

31 $$\frac{a}{{a}_{0}}=\frac{\varepsilon +{g}_{0}{(k-\mathrm{1)}}^{-{\delta }_{0}}}{\varepsilon +({g}_{0}+{g}_{1}M)(k-{\mathrm{1)}}^{-({\delta }_{0}+{\delta }_{1}M)}}\mathrm{.}$$

Since *ε* is a small number, we consider only the case of $\varepsilon \ll {g}_{0}{(k-\mathrm{1)}}^{-{\delta }_{0}}$ to get the approximated relative growth rate as

32 $$\frac{a}{{a}_{0}}\approx \frac{{g}_{0}}{{g}_{0}+{g}_{1}M}{(k-\mathrm{1)}}^{{\delta }_{1}M},$$

which can account for the *k*-dependence of *a*/*a*_0_, including the case with *α* = 1.5 in Fig. 4(e). In Fig. 4(e–g), we observe that the difference between curves of *a*/*a*_0_ for different *k*s increases and then decreases as *α* increases from 1.5 to 3. This non-monotonic behavior can be related to the non-monotonic behavior of *δ*_1_ as a function of *α*, as depicted in Fig. 5(f).

### Spreading in finite networks

So far we have focused on the spreading in Bethe lattices, i.e., regular networks of infinite size, which can also approximate the early-stage dynamics of spreading in finite networks as long as the cycles are rare. In addition to the early stage, the late-stage dynamics of spreading in finite networks has also been of interest^30,46^. For this, we employ two network models of size *N*: random regular graphs, in which every node has exactly *k* neighbors, and Erdös-Rényi random graphs, in which every possible pair of nodes is connected with a probability *p*, hence the average degree is *p*(*N*−1). On each of these graphs, both 2DSI and PSI dynamics are tested by the numerical simulations to measure the average numbers of infected nodes as a function of time, 〈*I*(*t*)〉. In all cases, we use networks of size *N* = 10^3^, and the results are averaged over 10^3^ simulation runs with different initial conditions for each parameter set.

**Figure 6:**
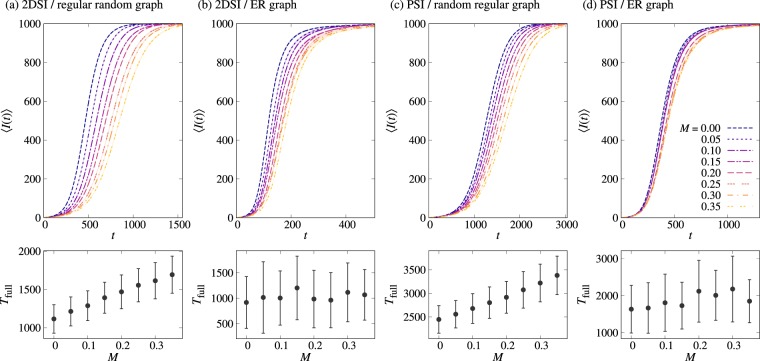
Top panels show the average numbers of infected nodes as a function of time, 〈*I*(*t*)〉, for the two-step deterministic SI dynamics (**a**,**b**) and the probabilistic SI dynamics with *η* = 0.1 (**c**,**d**) in two kinds of random networks of size *N* = 10^3^: regular random graphs with *k* = 4 (**a**,**c**) and Erdös-Rényi (ER) graphs with *p* = 0.008 (**b**,**d**). In all cases, we used *α* = 1.5. For each value of *M*, the average (dashed curve) and its standard error (shaded area) were obtained from 10^3^ runs with different initial conditions. Bottom panels show the average and standard deviation of the time it takes to fully infect the population, *T*_full_.

In Fig. 6 we find that the positive correlation between IETs lowers the average number of infected nodes for the almost entire range of time, except for the final-stage dynamics. This final-stage dynamics can be quantified by the time it takes to fully infect the population, denoted by *T*_full_. As depicted in the bottom panels of Fig. 6, the average value of *T*_full_ is clearly increasing with *M* when simulated in the regular random graphs, consistent with our previous results, while it appears to fluctuate around a constant in the case with ER graphs. Such fluctuating behavior of *T*_full_ in the case with ER graphs can be understood in terms of the variation of degrees of nodes, in contrast to the regular random graphs with a fixed degree for all nodes: At the final stage, it can take a longer time for some lower-degree susceptible nodes to get infected from their infected neighbors. This large timescale for the infection of low-degree nodes and its large fluctuations can eventually dominate over the early- and intermediate-stage dynamics that show clear dependence on *M*. In another work using the apparently power-law IET distributions without exponential cutoff^33^, the positive correlation between IETs was reported to reduce *T*_full_, calling for more systematic approaches.

## Conclusions

Spreading dynamics in temporal networks has been extensively studied for tackling the important issue of what features of temporal networks are most relevant to the speed of spreading taking place in such networks. One of the widely studied features is the inhomogeneity of interevent times (IETs), typically represented by heavy-tailed IET distributions, in the temporal interaction patterns between nodes. Although the impact of the inhomogeneous IETs on the spreading has been largely explored, yet little is known about the effects of correlations between IETs on the spreading. It is partly because many previous works have considered the contagion dynamics with the assumption of the immediate infection upon the first contact between susceptible and infected nodes, hence without the need to consider the correlated IETs. However, since temporal correlations in the interaction patterns can be fully understood both by IET distributions and by correlations between IETs^18^, the effects of inhomogeneous and correlated IETs on the spreading need to be systematically studied for better understanding the dynamical processes in complex systems. For this, we consider two contagion dynamics, i.e., two-step deterministic SI and probabilistic SI dynamics, naturally involving multiple consecutive IETs. For both dynamics, we derive analytical expressions of average transmission times 〈*r*〉 for a single-link setup, which turn out to linearly increase with the memory coefficient *M* as shown in equations (11) and (29). Therefore, the positive correlation between IETs is expected to slow down the spreading. By performing numerical simulations of the contagion dynamics in regular networks of infinite size and random graphs of finite size, we conclude that the positive correlation between IETs indeed slows down the spreading, compared to the case of uncorrelated IETs but from the same IET distributions.

The numerically obtained spreading speed, e.g., in Bethe lattices of degree *k*, could be successfully explained by means of the statistics of the shortest transmission time among *k* − 1 transmission times from one infected node to its *k* − 1 susceptible neighbors. In the case when IETs in the interaction patterns are largely homogeneous, the average transmission time 〈*r*〉 will serve as a representative timescale that determines the spreading speed. However, in the other case with inhomogeneous IETs or heavy-tailed IET distributions, the transmission time to each of *k* − 1 neighbors will be heterogeneously distributed, implying that neighbors infected earlier tend to spread the disease or information more quickly, hence more broadly, than those infected later. In this sense, the majority of the infected nodes can be largely explained by the descendants of early-infected neighbors, and the characteristic timescale of spreading speed can also be set by the average shortest transmission time 〈*r*_s_〉, rather than 〈*r*〉. Unfortunately, as the analysis of 〈*r*_s_〉 appears not to be straightforward, more detailed and rigorous understanding of the behavior of 〈*r*_s_〉 is left for future works.

So far we have focused on the positive memory coefficient based on the empirical observations, while the effects of the negative memory coefficient on spreading could also be studied. Our analytic results of average transmission times in equations (11) and (29) and numerical results on temporal networks enable us to predict the accelerated spreading due to the negativity of the memory coefficient. We also note that the shape of IET distributions may restrict the range of memory coefficient^42^, which should be properly considered when designing models.

Finally, we remark that in addition to the memory coefficient, the correlations between IETs have also been identified by other methods, e.g., in terms of bursty trains, which can detect long-range memory effects between IETs^5^: The number of events in each bursty train, i.e., the burst size, has been described by heavy-tailed distributions. Regarding this, the relation between memory coefficient and burst size distributions was recently studied^19^. Our approach can be extended by incorporating such heavy-tailed burst size distributions. We also note that more realistic network structures can be adopted for modeling temporal networks, such as networks with heterogeneous degrees^47^ and community structure^48^ among other network properties, e.g., stylized facts in social networks^49^.

## Methods

### Derivation of transmission time distributions

We first derive the transmission time distribution for the single-link transmission in the case with 1DSI. The transmission time *r* is equivalent to the time interval between a random arrival and the next event or contact. The probability that a random arrival falls within an IET of *τ* is *τP*(*τ*)/*μ*, where the mean IET *μ* is for the normalization. Given the arrival within the IET of *τ*, the probability of the transmission time being *r* is 1/*τ*. Then integrating such probability over the possible range of *τ* ≥ *r* gives the transmission time distribution as

33 $${Q}_{1{\rm{D}}}(r)={\int }_{r}^{\infty }\,d\tau \frac{\tau P(\tau )}{\mu }\frac{1}{\tau }=\frac{1}{\mu }{\int }_{r}^{\infty }\,d\tau P(\tau \mathrm{).}$$

The transmission time distribution for the 2DSI case can be derived similarly. Given the random arrival within an IET of *τ*_*i*_ and the residual time *r*_0_ to the next event, the transmission time *r* is *r*_0_ + *τ*_*i*+1_. The probability of having *τ*_*i*+1_ is given by the conditional distribution *P*(*τ*_*i*+1_|*τ*_*i*_), i.e., *P*(*r* − *r*_0_|*τ*_*i*_). Together with the probabilities we already have for the 1DSI case, we obtain the transmission time distribution for the 2DSI case by integrating the probability over *τ*_*i*_ ≥ *r*_0_ and 0 ≤ *r*_0_ ≤ *r* as

34 $${Q}_{2{\rm{D}}}(r)={\int }_{0}^{r}\,d{r}_{0}{\int }_{{r}_{0}}^{\infty }\,d{\tau }_{i}\frac{{\tau }_{i}P({\tau }_{i})}{\mu }\frac{1}{{\tau }_{i}}P(r-{r}_{0}|{\tau }_{i})=\frac{1}{\mu }{\int }_{0}^{r}\,d{r}_{0}{\int }_{{r}_{0}}^{\infty }\,d{\tau }_{i}P({\tau }_{i})P(r-{r}_{0}|{\tau }_{i}\mathrm{).}$$

The last expression is rewritten by introducing the integration with respect to *τ*_*i*+1_:

35 $${Q}_{2{\rm{D}}}(r)=\frac{1}{\mu }{\int }_{0}^{r}\,d{r}_{0}{\int }_{0}^{\infty }\,d{\tau }_{i+1}{\int }_{{r}_{0}}^{\infty }\,d{\tau }_{i}P({\tau }_{i},{\tau }_{i+1})\delta (r-{r}_{0}-{\tau }_{i+1}),$$

which then can be straightforwardly extended to the more general case for the transmission time after *l* failed attempts, see equation (23).

### Derivation of the average transmission time after *l* failed attempts

The average of the transmission time after *l* failed attempts, i.e., 〈*r*_*l*_〉 in equation (24) can be calculated using *Q*_*l*_(*r*) in equation (23) as follows:

36 $$\langle {r}_{l}\rangle =\frac{1}{\mu }{\int }_{0}^{\infty }\,drr{\int }_{0}^{r}\,d{r}_{0}\prod _{j=1}^{l}{\int }_{0}^{\infty }\,d{\tau }_{i+j}{\int }_{{r}_{0}}^{\infty }\,d{\tau }_{i}P({\tau }_{i},\,\cdots ,\,{\tau }_{i+l})\delta (r-{r}_{0}-\sum _{j=1}^{l}\,{\tau }_{i+j})\mathrm{.}$$

We interchange the order of integration with respect to *r*_0_ and *τ*_*i*_, i.e.,

37 $${\int }_{0}^{r}\,d{r}_{0}{\int }_{{r}_{0}}^{\infty }\,d{\tau }_{i}={\int }_{0}^{\infty }d{\tau }_{i}{\int }_{0}^{{\rm{\min }}\,\{r,{\tau }_{i}\}}\,d{r}_{0}$$

to rewrite equation (36) as

38 $$\langle {r}_{l}\rangle =\frac{1}{\mu }\prod _{j=0}^{l}{\int }_{0}^{\infty }\,d{\tau }_{i+j}P({\tau }_{i},\,\cdots ,\,{\tau }_{i+l}){\int }_{0}^{\infty }\,drr{\int }_{0}^{{\rm{\min }}\,\{r,{\tau }_{i}\}}\,d{r}_{0}\delta (r-{r}_{0}-\sum _{j\mathrm{=1}}^{l}\,{\tau }_{i+j})\mathrm{.}$$

Integration with respect to *r* and *r*_0_ in the above equation is straightforward:

39 $${\int }_{\sum _{j\mathrm{=1}}^{l}{\tau }_{i+j}}^{\sum _{j\mathrm{=0}}^{l}{\tau }_{i+j}}drr=\frac{{\tau }_{i}^{2}}{2}+{\tau }_{i}\sum _{j\mathrm{=1}}^{l}\,{\tau }_{i+j},$$

enabling us to finally obtain

40 $$\langle {r}_{l}\rangle ={\langle r\rangle }_{1{\rm{D}}}+\frac{1}{\mu }\sum _{j=1}^{l}\,\langle {\tau }_{i}{\tau }_{i+j}\rangle \mathrm{.}$$

Note that 〈*r*〉_1D_ = 〈*τ*^2^〉/(2*μ*) in equation (5), with 〈*τ*^2^〉 denoting the second moment of the IET distribution *P*(*τ*).

## Data Availability

The data generated and analysed in this study are available from the authors on request.
